# Creation of a universal language for surgical procedures using the step‐by‐step framework

**DOI:** 10.1002/bjs5.47

**Published:** 2018-04-27

**Authors:** T. Nazari, E. J. Vlieger, M. E. W. Dankbaar, J. J. G. van Merriënboer, J. F. Lange, T. Wiggers

**Affiliations:** ^1^ Incision Academy Amsterdam The Netherlands; ^2^ Department of Surgery Erasmus University Medical Centre Rotterdam The Netherlands; ^3^ Institute of Medical Education Research Rotterdam and Department of Education Erasmus University Medical Centre Rotterdam The Netherlands; ^4^ Department of Educational Development and Research, Faculty of Health, Medicine and Life Sciences Maastricht University Maastricht The Netherlands

## Abstract

**Background:**

Learning of surgical procedures is traditionally based on a master–apprentice model. Segmenting procedures into steps is commonly used to achieve an efficient manner of learning. Existing methods of segmenting procedures into steps, however, are procedure‐specific and not standardized, hampering their application across different specialties and thus worldwide uptake. The aim of this study was to establish consensus on the step‐by‐step framework for standardizing the segmentation of surgical procedures into steps.

**Methods:**

An international expert panel consisting of general, gastrointestinal and oncological surgeons was approached to establish consensus on the preciseness, novelty, usefulness and applicability of the proposed step‐by‐step framework through a Delphi technique. All statements were rated on a five‐point Likert scale. A statement was accepted when the lower confidence limit was 3·00 or more. Qualitative comments were requested when a score of 3 or less was given.

**Results:**

In round one, 20 of 49 experts participated. Eighteen of 19 statements were accepted; the ‘novelty’ statement needed further exploration (mean 3·05, 95 per cent c.i. 2·45 to 3·65). Based on the qualitative comments of round one, five clarifying statements were formulated for more specific statements in round two. Twenty‐two experts participated and accepted all statements.

**Conclusion:**

The international expert panel consisting of general, gastrointestinal and oncological surgeons supported the preciseness, usefulness and applicability of the step‐by‐step framework. This framework creates a universal language by standardizing the segmentation of surgical procedures into step‐by‐step descriptions based on anatomical structures, and may facilitate education, communication and assessment.

## Introduction

Throughout history, surgical procedures have been taught in a master–apprentice model^1^. The apprentice starts by observing the master performing a surgical procedure, and he or she is gradually allowed to take over parts of the procedure until he or she can perform the entire surgery without supervision. This model relies on the ability of expert surgeons to provide a demonstration with clear explanation of surgical procedures, but research has shown that information regarding the surgery becomes self‐evident for experts. Consequently, they unintentionally omit critical information when explaining a procedure to apprentices^2^.

Decreased teaching time due to regulation of working hours and the emphasis on operating room efficiency has made it more challenging to teach procedures to surgical residents (trainees)^3^. The priority should therefore be on learning before setting foot in the operating room. When residents are preparing for procedures, many sources are available to them, such as books, articles and videos. Often these sources describe or demonstrate the surgical procedures as a continuous text or video. Different step‐by‐step descriptions of the same procedure can vary greatly in terms of steps, structures, components and demarcations.

To facilitate the learning process, the cognitive limitations of learners must be taken into account. Cognitive learning theory explains the mechanism of processing and storing new information. When information is seen or heard, it can be actively processed in working memory by connecting it to existing knowledge in the long‐term memory, and then be stored in long‐term memory as cognitive schemas. This processing and storing of information is bound by a limited cognitive capacity^4^. To use this capacity optimally, complex transient information (such as video or animation) should be presented in segments rather than as a continuous stream^5^. Segmentation divides information into smaller pieces with pauses in between that provide the learner time to process one segment before moving on to the next segment. In addition, segmentation could aid in constructing a cognitive schema representing the procedure. This works especially for novices as they do not yet possess the cognitive schemas necessary to understand a newly presented procedure quickly^6^.

To create a universal language of surgical procedures that keeps the segmentation principle in mind, a generic framework should be designed. The definition of a framework is ‘a structure underlying “something” serving a specific purpose’^7^, or as the *Oxford Living Dictionary* defines it: ‘a basic structure underlying a system, concept, or text’^8^. This framework should clearly describe how to structure a surgical description and where to segment.

Previous attempts at creating a generic framework have not yet been implemented universally. The definitions used for the steps and other elements in the previous frameworks were unclear and ambiguous^9^ ^10^, decreasing the reproducibility and transferability of these frameworks. Other described procedure‐specific methods were time‐intensive, as the steps were derived from expert panels^11^ ^12^ or after video‐recording and observing multiple operations^13^. The segmentation of surgical procedures is lacking a unified and standardized approach.

In this article, one generic framework is proposed to structure the segmentation of surgical procedures into steps and substeps. This framework could create one common language of surgical procedures for daily use among surgeons and surgical residents. It would offer a foundation for surgical education concerning descriptions of surgical procedures in books, courses and articles. The aim of this study was to assess whether international experts agree on the preciseness, novelty, usefulness and applicability of a newly developed step‐by‐step framework to segment surgical procedures into steps.

## Methods

### The step‐by‐step framework

**Figure 1:**
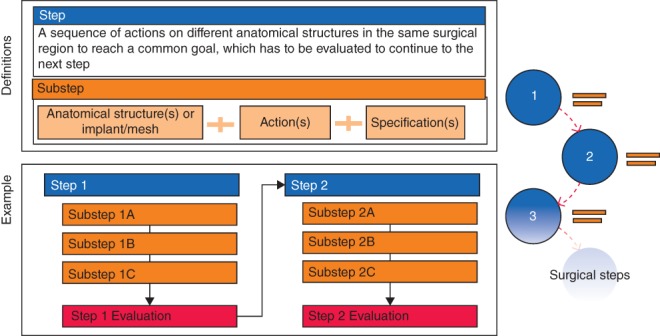
The step‐by‐step framework. Each step consists of one or more substeps and must be evaluated before continuing to the next step. A substep is a combination of an anatomical structure or implant with an action and specification

The proposed step‐by‐step framework structures the description and demonstration of surgical procedures. This framework is not procedure‐specific and aims to be broadly applicable to all surgical disciplines. Within the framework, steps and substeps are defined based on the anatomical structures encountered during the described procedure (*Fig*. 1). A step is performed in one surgical region to reach a predetermined goal that has to be evaluated before continuing to the next step. Each step is named according to its goal. By doing so, the surgery is broken down into meaningful events. A step consequently consists of substeps.

A substep is based on an anatomical structure or implant, such as a mesh or osteosynthesis material, and contains one or more actions. The description of every action is a single verb (such as transect or incise). If the predetermined goal of a step has not been achieved, the course of its substeps has to be reviewed and revised until the goal is reached.

In the specification of a substep, an explanation is provided for the combination of an anatomical structure with an action. A specification containing hazardous parts is called ‘hazards’, and suggestions for the learner's convenience are ‘tips’.

### Delphi method

A Delphi method was used to establish consensus on the step‐by‐step framework. The Delphi method is used to gather anonymous feedback of an expert panel via questionnaires with statements in order to establish group consensus^14^. The experts scored their agreement with each statement, in this case on a five‐point Likert scale^15^. The responses are then analysed and the statements are either accepted, rejected or further explored by additional statements, until group consensus is achieved.

#### *Expert panel*

An international panel was selected with the main objective that the surgeons were responsible for the training and education of surgical residents. Special attention was paid to include different disciplines (general, oncological and gastrointestinal) and global spread in both high‐, middle‐ and low‐resource settings (*Table* 1). The surgeons were not approached before the Delphi rounds. The online questionnaires were sent directly via e‐mail.

**Table 1:** Country of origin of experts

	Total (*n* = 49)	Round one (*n* = 20)	Round two (*n* = 22)
Europe			
Austria	1		1
Bulgaria	1		
Denmark	2		
France	3	3	
Georgia	1	1	1
Germany	1	1	1
Italy	3	2	1
The Netherlands	9	2	6
Poland	4	2	1
Russia	1	1	1
Slovenia	1		1
Spain	1		1
Sweden	1		
UK	7	2	2
Africa			
Ghana	5	2	2
South Africa	1		
South America			
Brazil	2	2	
Central America			
Curacao	2	1	2
North America			
USA	2		1
Asia			
Afghanistan	1	1	1

An anonymous questionnaire facilitates decision‐making as individuals might be more open and honest with their ratings^16^. The Delphi rounds, therefore, were completely anonymous and all experts were approached for the second round. Each round lasted 2 weeks, during which the experts were reminded twice via e‐mail.

#### *Round one*

As the framework was developed before commencing the Delphi method, the first 19 statements were constructed based on the step‐by‐step framework, its characteristics and applicability. The statements were in the following categories: preciseness (9 items), novelty (1 item), usefulness (3 items), and applicability (6 items) (*Table* 2; *Tables S1* and *S2*, supporting information). The applicability was tested with the steps of open inguinal hernia repair and open small bowel resection. Each statement was rated on a five‐point Likert scale (1, strongly disagree; 5, strongly agree). The experts were requested to give qualitative comments when a score of 3 or less was given, although comments were not mandatory.

**Table 2:** Statements in round one

	Statement		Mean
Preciseness (Cronbach's α = 0·862)	1	Each surgical procedure is constructed of multiple steps that have to be performed	4·45 (4·09, 4·81)
	2	Alternative routes within one procedure are possible	4·25 (3·75, 4·75)
	3	Each surgical step starts with determining the wanted goal	4·65 (4·42, 4·88)
	4	To accomplish the determined goal, one or more structures are encountered and dealt with	4·60 (4·36, 4·84)
	5	Each step ends with evaluation of whether the common predetermined goal has been achieved	4·65 (4·42, 4·88)
	6	To accomplish the common predetermined goal of a step, one or more smaller steps have to be taken	4·40 (4·02, 4·78)
	7	The substep is a combination of one structure with one or more actions	4·35 (3·97, 4·73)
	8	A substep is based on an anatomical structure or an implant	4·40 (4·12, 4·68)
	9	A substep can be based on non‐anatomical structures, such as meshes and implants	4·50 (4·26, 4·74)
Novelty	10	The proposed step‐by‐step concept is new in the surgical world*	3·05 (2·45, 3·65)
Usefulness (Cronbach's α = 0·830)	11	The proposed step‐by‐step concept is useful in communication with other surgeons	4·55 (4·31, 4·79)
	12	The proposed step‐by‐step concept is useful in research concerning surgical procedures	4·55 (4·31, 4·79)
	13	The proposed step‐by‐step concept is useful in teaching surgeons in training	4·75 (4·54, 4·96)
Applicability (Cronbach's α = 0·877)	Tested on the steps of open inguinal hernia repair		
	14	The steps represent the natural moments of evaluation during surgery	4·45 (4·21, 4·69)
	15	The start of this step is a natural moment to determine a new goal during the surgery	4·55 (4·31, 4·79)
	16	The end of this step is a natural evaluation moment before moving on to the next step during the surgery	4·60 (4·36, 4·84)
	Tested on the steps of open small bowel resection		
	17	The steps represent the natural moments of evaluation during surgery	4·15 (3·80, 4·50)
	18	The start of this step is a natural moment to determine a new goal during the surgery	4·35 (4·08, 4·62)
	19	The end of this step is a natural evaluation moment before moving on to the next step during the surgery	4·30 (3·99, 4·61)

Values in parentheses are 95 per cent confidence intervals.

* Statement in need of further exploration.

Each statement was analysed to determine whether it was accepted or not. Statistical analysis was based on the findings of a previous study^12^. For each statement a score between 1·00 and 5·00 was possible. The mean with the 95 per cent c.i. was calculated per statement. A statement was accepted if the lower confidence limit was 3·00 or more. A statement was rejected if the upper confidence limit was less than 3·50. A statement was further explored in the second round when it did not meet the above‐mentioned criteria. The internal consistency per category was determined using Cronbach's α; preciseness (9 items), usefulness (3 items) and applicability (6 items). The analyses were performed using the SPSS® version 24.0 (IBM, Armonk, New York, USA).

#### *Round two*

As the responders could not be identified from the non‐responders in the anonymous online questionnaire, the questionnaire for round two was sent to all experts. Any statements from the first round that were not accepted or rejected were explored further, leading to new statements for the next round. These statements were based on the qualitative comments that were gathered in the first round. The new statements were sent out in the second round. These were scored and analysed in the same manner as those in round one.

## Results

### Expert panel

Forty‐nine expert surgeons, of whom 22 were full professors in surgery, were invited to participate. Of these 49 experts, 20 replied and assessed the statements in round one. In the second round, these statements were again sent to all 49 experts, of whom 22 replied and assessed the statements.

### Round one

In the first round, the experts originated from 12 different countries and four different continents (*Table* 1). Fifteen experts had more than 20 years postresidency experience, three had 10–20 years, and two had up to 10 years. Of the 19 statements, 18 were accepted and one needed further exploration (*Table* 2). The internal consistency for preciseness was Cronbach's α = 0·862, usefulness Cronbach's α = 0·830 and applicability Cronbach's α = 0·877.

### Round two

One statement from the first round was in need of further exploration: ‘The proposed step‐by‐step concept is new in the surgical world’. The qualitative comments on this statement concerning novelty were analysed. Of the ten gathered comments, five concerned its preciseness. The comments were divided into two categories: ‘not new’ and ‘not new, but now defined’ (*Table* 3). The emphasis in the second round shifted from ‘novelty’ in the surgical world to ‘preciseness’ (*Table* 4). The experts were approached again. In the second round, 22 experts of the 49 approached participated. All statements were accepted.

**Table 3:** Qualitative comments on statement: ‘The proposed step‐by‐step concept is new in the surgical world’

Not new	Not new, but now defined
‘Several old books present step‐by‐step procedures, e.g. Zollinger’	‘The concept is not new, but now it seems to be properly evaluated, appreciated, and defined’
‘In the description of procedures, the step‐by‐step approach is sometimes used’	‘See my published research on INVEST for lap cholecystectomy, however, this is the first research to accurately define the step/substep concept’
‘The consideration step‐by‐step procedure in surgery has always been respected since long time ago’	‘This is how I was taught, and have been teaching. The steps were however not always strictly defined’
‘This has been known for years’	‘It may not be universal in practice but is highly recommended for standardization and audit’
‘I am not sure it's entirely new’	‘A procedure is always a progression. I don't see really what is new except a formalization surgery by surgery which could help to establish standard report for example’

**Table 4:** Statements in round two

		Formulated statements	Mean
Existing step‐by‐step descriptions	1	Describing surgeries in steps exists in the surgical world	4·09 (3·71, 4·48)
Preciseness	2	Describing a surgical step is procedure‐specific as the goals vary between the different surgeries	4·23 (3·99, 4·46)
	3	Describing a substep is generic and interchangeable between surgeries as it is based on anatomical structures or implants combined with one or multiple actions*	4·00 (3·66, 4·34)
	4	Describing substeps based on anatomical structures or implants combined with one or multiple actions is relevant	4·45 (4·23, 4·68)
	5	Describing substeps based on actions from a predefined list is an improved addition in the step‐by‐step concept	4·32 (4·11, 4·53)

Values in parentheses are 95 per cent confidence intervals.

* For example, the substep ‘transect greater saphenous vein’ (combination of an action and an anatomical structure) occurs in more than one surgery.

## Discussion

In this study, a framework to segment surgical procedures into a uniform and standardized method was proposed. The framework aimed to be broadly applicable to all surgical disciplines. It was presented to an international expert panel consisting of general, gastrointestinal and oncological surgeons to assess their agreement on its preciseness, novelty, usefulness and applicability. In the first round, one statement concerning novelty needed further exploration: ‘The proposed step‐by‐step concept is new in the surgical world’. The original statement from round one was rephrased in five clarifying statements exploring different aspects of the presented step‐by‐step framework. The focus of the statements shifted from ‘novelty’ to ‘preciseness’. The panel established consensus in two Delphi rounds on the preciseness, usefulness and applicability of the framework. Consensus was not achieved on the novelty of the step‐by‐step framework. The use of substeps, being based on anatomical structures with a predefined list of subsequent actions, was found to be an improvement in the framework.

The novelty of the framework was not agreed upon. Segmenting surgical procedures into steps and substeps is not new in the surgical world, as old books described procedures in a step‐by‐step manner. Previously published studies have described their own method of segmenting surgical procedures, but these have not yet been implemented widely. One explanation might be that the previous frameworks were unclear and ambiguous in the definitions used for the steps and other elements^9^ ^10^. Another explanation may be the time‐intensive process of defining the steps. Sarker and colleagues^13^ described a method that used a surgical task analysis to construct a surgical description. This method consisted of reviewing literature and textbooks, expert panel discussions and video‐recordings. It is thorough, but time‐consuming. Furthermore, it is applicable only to one surgery. Other studies performed video analyses^10^ ^17^, ^18^ or expert panel discussions^11^ ^12^, ^19^ to segment one surgery.

Even though step‐by‐step descriptions are not new, the clear and unambiguous defining within a framework is. In particular, the definition of substeps being based on anatomical structures and using a predefined list of subsequent actions was seen as an improvement in the step‐by‐step framework. With the step‐by‐step framework, the steps can be defined for all surgical procedures in a unified and time‐efficient manner. The steps of different operations segmented by this framework are generic and compatible with one another. This has many practical implications. The international expert panel supported the usefulness of the step‐by‐step framework. It may facilitate the education of surgeons and other surgical staff, communication between all surgical staff, and the assessment of surgeons (in training).

The segmenting principle of cognitive learning theory was applied to the step‐by‐step framework to facilitate education. Efficiency of learning is higher when prior knowledge can be referred to for creating new knowledge^4^. Within surgery, anatomical knowledge is fundamental, so anatomy is the basis of the proposed step‐by‐step framework^6^.

The step and substep definitions of the step‐by‐step framework were assessed as clear, concise and unambiguous by the international expert panel. All aspects of a surgical procedure can be fitted into the framework. The definitions are mutually exclusive and collectively exhaustive. With the step‐by‐step framework, segmentation of surgical procedures into steps can be performed in a unified and standardized manner, creating one universal language for surgical procedures.

The framework can structure and standardize information transfer and communication between surgical staff regarding surgical procedures; this may lead to improved surgical safety^20^. Standardized and structured methods to improve surgical safety are widely used and effective. For instance, implementation of the surgical safety checklist has led to a significant reduction in postoperative complications and death rates^21^ ^22^. Furthermore, van de Graaf and co‐workers^23^ defined key moments during colorectal surgery, which were video‐recorded to augment the traditional written synoptic operation reports. The operative notes demonstrated improved availability of essential information when the videos were combined with the traditional reports. One of the reasons suggested by the authors was the stepwise approach employed during systematic video registration.

Assessment of surgeons can be performed using checklists, such as the commonly used Objective Structured Assessment of Technical Skills (OSATS)^24^. OSATS uses an operation‐specific method to assess a surgeon. A surgery‐specific method is the Observational Clinical Human Reliability Analysis (OCHRA), which assesses each surgical step for procedural and executional errors. Procedural errors occur when a step is not performed in the correct order or is omitted entirely, whereas executional errors contain the technical errors made within a step^25^ ^26^. To use OCHRA adequately to assess a procedure, the surgical steps have to be defined in a standardized, generic and time‐efficient manner, which can be accomplished with the step‐by‐step framework. The steps of different surgical procedures segmented by the step‐by‐step framework are compatible, facilitating the assessment of a surgeon. In a training environment, this method provides the trainee with specific feedback for each step of the procedure.

This was a study with an international expert panel including surgeons from countries with high‐, middle‐ and low‐resource settings. The framework was found to be applicable in all of these settings, as expected, because the main emphasis of the framework is on anatomical structures, not equipment or materials.

The experts were completely anonymous during this study, facilitating decision‐making, as individuals might then be more open and honest with their ratings^16^. This anonymity, however, meant that the round two questionnaires had to be sent to all expert surgeons (not only those who participated in round one), resulting in participants in the second round who had not participated in the first.

In this study, experts in general, gastrointestinal and oncological surgery were included, and the number of procedures assessed during the rounds was limited to two. In further research, other fields of surgery should be included, as this framework should be applicable to any surgical specialty. Participants in further research should be able to test the framework on their own procedures in a training setting.

Even though two reminders were sent out per round, the participation rate of 20 of 49 in the first round and 22 of 49 in the second round was lower than anticipated. The first reason might be that the experts were not approached before conducting the research. In addition, this Delphi method was carried out as an online questionnaire, notorious for low response rates^27^.

The step‐by‐step framework is suitable for the standardized segmentation of surgical procedures into a uniform step‐by‐step description and demonstration. This framework creates a universal language of surgical procedures that may facilitate education, communication and assessment.

## Disclosure

The authors declare no conflict of interest.

## Supporting information


**Table S1.** Step‐by‐step description of open inguinal hernia repair
**Table S2.** Step‐by‐step description of open small bowel resection
