# Supplementary material for: Creation of a universal language for surgical procedures using the step‐by‐step framework
Source: BJS Open. 2018 Apr 27;2(3):151–7. doi: 10.1002/bjs5.47 (PMC5989977; doi:10.1002/bjs5.47)
Supplement: Supplementary file 1 — Table S1. Step‐by‐step description of open inguinal hernia repair Table S2. Step‐by‐step description of open small bowel resection [file BJS5-2-151-s001.docx]

**BJS5_47**

**Creation of a universal language for surgical procedures using the step-by-step framework**

**T. Nazari, E. J. Vlieger, M. E. W. Dankbaar, J. J. G. van Merriënboer, J. F. Lange and T. Wiggers**

| **Table S1** Step-by-step description of open inguinal hernia repair | | | |
| --- | --- | --- | --- |
| **Step** | **Substep** | **Action** | **Specification** |
| 1. External oblique aponeurosis exposure | A. Skin | Anesthetize | Anesthetize the skin with a subdermal infiltration of 5cc of anesthetic mixture along the line of the planned incision parallel to the surface of the skin. |
|  |  | Anesthetize | Anesthetize the skin intradermal by withdrawing the needle until the tip of the needle reaches the intradermic level and slowly injecting (3cc) along the line of the intended incision. |
|  |  |  | TIP - Local anaesthesia A 50:50 mixture of 1% lidocaine and 0.5% bupivacaine is recommended, 45 cc should (on average) be sufficient for a unilateral hernia repair. Adding epinephrine can prolong anesthetic duration time further and decrease toxicity of the local anesthetic due to its slower absorption. Infiltrate with a moving needle, this minimizes the risk of prolonged intravenous infiltration. |
|  | B. Subcutaneous tissue | Anesthetize | Anesthetize the deep subcutaneous tissue (10 cc) by vertically inserting the needle perpendicular to the skin surface, 2 cm apart along the planned incision line. |
|  | C. Skin | Incise | Incise the skin for a length of approximately 5-8 cm in the line between anterior superior iliac spine to the pubic tubercle. For adequate exposure, the incision should be placed directly over the pubic tubercle. An incision placed in the Langer’s skin lines, is a more cosmetic option. |
|  | D. Subcutaneous tissue | Incise | Incise the subcutaneous tissue until Scarpa’s fascia is reached. |
|  |  |  | HAZARD - Superficial inferior epigastric vessels damage During the skin incision caution should be taken for the superficial inferior epigastric vessels. Insufficient ligation of these veins can result in postoperative haemorrhage and it is recommended to ligate this vein. |
|  | E. Superficial inferior epigastric vein | Transect | Transect the superficial inferior epigastric vein. |
|  | F. Scarpa's fascia | Incise | Incise Scarpa’s fascia to expose the external oblique aponeurosis. |
|  | G. External oblique aponeurosis | Anesthetize | Anesthetize the subfascial level (8-10 cc) by injecting immediately underneath the aponeurosis of the external oblique muscle, to sedate the ilioinguinal, iliohypogastric and genitofemoral nerve and separate the external oblique aponeurosis from the underlying ilioinguinal nerve. |
|  |  |  |  |
| 2. Inguinal canal exposure | A. External oblique aponeurosis | Incise | Incise the aponeurosis of the external oblique muscle over a length of 3-4 mm in the direction of the fibres. Extend the incision toward the external inguinal ring medially. |
|  | B. Ilioinguinal nerve | Identify | Identify the ilioinguinal nerve on top of the spermatic cord, which can be done after opening the aponeurosis. |
|  |  | Preserve | Preserve the ilioinguinal nerve. |
|  |  |  | HAZARD - Ilioinguinal nerve damage While opening the aponeurosis of the external oblique muscle, care should be taken for the ilioinguinal nerve. This nerve is located directly underneath the aponeurosis of the external oblique muscle. Sometimes, it is even possible to see this nerve through the thin aponeurosis. |
|  | C. External oblique aponeurosis | Extend | Extend the incision in the aponeurosis of the external oblique muscle towards 3 cm lateral to the internal inguinal ring. |
|  |  | Dissect | Dissect the external oblique aponeurosis by developing the plane between the aponeurosis of the external oblique muscle and the internal oblique muscle caudally, while avoiding the ilioinguinal nerve and the iliohypogastric nerve. |
|  | D. Iliohypogastric nerve | Identify | Identify the illiohypogastric nerve. |
|  |  | Preserve | Preserve the iliohypogastric nerve. |
|  |  |  | HAZARD - Iliohypogastric nerve damage After opening the aponeurosis of the external oblique muscle, the iliohypogastric nerve can be found lying on the internal oblique abdominal muscle after the edges of the external oblique aponeurosis are elevated. Failure to identify this nerve, may result in damage of this nerve which may result in chronic pain. |
|  | E. Pubic tubercle | Anesthetize | Anesthetize with a local anesthetic at the level of the pubic tubercle where the rectus sheath is attached. |
|  |  |  |  |
| 3. Spermatic cord mobilization | A. Genital branch of the genitofemoral nerve | Identify | Identify the ‘blue line’, which points out the external spermatic vein and is kept with the spermatic cord, ensuring the genital branch of the genitofemoral nerve that runs along the ‘blue line’ is protected during the procedure. |
|  |  | Preserve | Preserve the genital branch of the genitofemoral nerve. |
|  | B. Spermatic cord | Isolate | Isolate the spermatic cord completely from the floor of the inguinal canal and the pubic bone, 2 cm medial of the pubic tubercle. |
|  |  | Encircle | Encircle the spermatic cord with a Penrose drain. |
|  |  |  |  |
| 4. Hernia sac removal | A. External spermatic fascia | Identify | Identify the external spermatic fascia with the junction of the external spermatic and the deep inferior epigastric vessels on the lateral side. If a lipoma of the cord is present, it should be excised. |
|  |  | Incise | Incise the external spermatic fascia. |
|  | B. Cremaster muscle | Split | Split the cremaster muscle fibres. |
|  |  |  | TIP Large hernia  In large hernias separation of the cremaster muscle fibres is usually not possible. In these cases, the fibres are transected. Either the peritoneal fold is identified to rule out an indirect hernia, or the (indirect) hernia sac is separated from the spermatic cord. It is always necessary to exclude an indirect hernia. |
|  | C. Hernia sac | Identify | Identify the hernia sac. If neither a direct nor an indirect hernia is found, the diagnosis of a previously unrecognized femoral hernia should be considered. |
|  |  | Anesthetize | Anesthetize the hernia neck or hernia sac by infiltrating it with an anesthetic. |
|  |  | Remove | Remove the hernia sac. In a direct hernia reduce through the posterior wall. In a large hernia ligate and transect the hernia sac. Avoid using sutures. |
|  |  |  |  |
| 5. Mesh placement | A. Inguinal canal | Expose | Expose the lower edge of the inguinal canal, the inguinal ligament. |
|  | B. Mesh | Trim | Trim the mesh to fit the inguinal canal. |
|  |  | Fixate - medial | Fixate the mesh medially to the distal anterior rectus sheath with 2 cm overlap over the pubic bone. |
|  |  |  | HAZARD - Pubic periosteum damage During medial fixation of the mesh to the anterior rectus sheath, care should be taken not to include the pubic periosteum in this suture as this may result in chronic pain. |
|  |  | Fixate - caudal | Fixate caudally by continuing with a running suture on the lower edge of the inguinal ligament until the level of the internal inguinal ring in 4 to 5 steps. Do not continue this suture beyond the lateral border of the internal inguinal ring. |
|  |  |  | HAZARD - Femoral vessels and nerve damage During the caudal fixation, avoid damaging the femoral vessels and nerve which run just underneath the inguinal ligament. Damage is avoided by only taking small bites of the lower edge of the inguinal ligament (1-2 mm). |
|  |  | Split | Split the mesh from lateral to medial until the medial border of the internal inguinal ring, creating two tails. The superior tail is approximately 2/3 of the width of the mesh and the inferior tail is approximately 1/3 of the width of the mesh. |
|  |  |  | TIP - Level of the new internal inguinal ring Release the Penrose drain to lower the spermatic cord and to be able to determine where the level of the new internal inguinal ring should be. |
|  |  | Position | Position the mesh under the spermatic cord, and subsequently manoeuvre the spermatic cord between the two tails. Then, pass the superior tail over the inferior tail to create a prosthetic internal inguinal ring. |
|  |  |  | TIP - Spermatic cord passage Create a prosthetic internal inguinal ring wide enough for the spermatic cord to pass through. Avoid making the prosthetic ring too wide, as a recurrent hernia might develop. A forceps inserted in the prosthetic ring should be able to separate 2-3 mm. If the ring is too wide, use a non-absorbable suture to reduce the prosthetic ring medially. If the ring is too tight, extend the cut using scissors. |
|  |  | Fixate - lateral | Fixate both tails with a single non-absorbable suture to the inguinal ligament. |
|  |  | Trim | Trim the tails on the lateral side. |
|  |  | Position | Position the tails of the mesh under the external oblique aponeurosis laterally and cranially. |
|  |  | Fixate - cranial | Fixate the superior margin of the mesh with one or two interrupted absorbable sutures to the aponeurosis of the internal oblique muscle, while avoiding the iliohypogastric nerve. |
|  |  |  | HAZARD - Iliohypogastric nerve damage Avoid damage or entrapment of the iliohypogastric nerve, preferably by identification. Make a transverse suture rather than a longitudinal suture, as this minimizes the risk of nerve entrapment. If necessary a separate cut can be made into the mesh to free the iliohypogastric nerve. If the nerve cannot be freed from the mesh, transect this nerve and bury the iliohypogastric nerve in the internal oblique muscle. |
|  |  |  | HAZARD - Ilioinguinal nerve entrapment Do not fixate the superior margin of the mesh lateral to the internal inguinal ring. The ilioinguinal nerve runs just beneath the oblique internal muscle. Sutures could result in entrapment of this nerve, which could result in chronic pain. |
|  |  |  |  |
| 6. Wound closure | A. Drain | Remove | Remove the Penrose drain under the spermatic cord. |
|  | B. External oblique aponeurosis | Close | Close the aponeurosis of the external oblique muscle anterior to the cord with a slow-absorbable running suture, creating a new external ring. |
|  | C. Subcutaneous tissue | Close | Close the subcutaneous plane including Scarpa’s fascia with 3-4 standing absorbable sutures. |
|  | D. Skin | Close | Close the skin intracutaneously with a monofilament absorbable suture. |
|  |  | Anesthetize | Anesthetize the skin with a local postoperative anesthetic to reduce postoperative pain when the procedure is not performed under local anaesthesia. |

| **Table S2** Step-by-step description of open small bowel resection | | | |
| --- | --- | --- | --- |
| **Step** | **Substep** | **Action** | **Specification** |
| 1. Abdominal cavity approach | A. Skin | Incise | Incise the skin in the midline from the umbilicus to the pubic bone. |
|  |  |  | TIP Incision scarring To minimize postoperative scarring, the skin incision is placed preferably over old incision scars. |
|  | B. Subcutaneous tissue | Incise | Incise the subcutaneous tissue until the linea alba is encountered. |
|  | C. Linea alba | Incise | Incise the linea alba. |
|  |  |  | TIP - Linea alba Identification of the linea alba, is facilitated by equal lateral traction on the skin and subcutaneous tissue. The linea alba is formed by fusion of contralateral aponeuroses of the abdominal muscles, therefore it can be identified by looking for a white line of crossing fibres. |
|  | D. Peritoneum | Identify | Identify the peritoneum as a thin smooth layer usually with the presence of preperitoneal fat. |
|  |  | Lift | Lift the peritoneum to prevent damage to the intraperitoneal organs. |
|  |  | Incise | Incise the peritoneum with scissors and be careful not to damage the posteriorly-lying intraperitoneal organs. |
|  |  |  | TIP - Peritoneal opening Opening of the peritoneum should be performed in a section without prior surgery. Here the chances of adhesions will be small and the risk of intraperitoneal organ injury minimized. |
|  |  |  |  |
| 2. Mesentery transection | A. Bowel | Identify | Identify the transection site. Important is (1) if the bowel looks well vascularized, (2) the two ends can be brought together tension free, and (3) if the lumen sizes are comparable. |
|  |  |  | TIP - Lumen size difference If the difference in size is too big to correct during suturing you can choose to either go for an End to Side or Side to Side anastomosis, or enlarge one end by cutting in an oblique way from the antimesenteric to the mesenteric side until it matches the other lumen. Another option is to make a dorsal slit on the antimesenteric side of the part with the smallest lumen. |
|  | B. Visceral peritoneum | Incise | Incise the visceral peritoneum covering the mesentery, from the determined sites to central in a V-shape. |
|  |  |  | TIP - Location intestinal branches.  Intestinal branches can be located with transillumination and after the incision of the peritoneum. |
|  | C. Mesentery | Transect | Transect the mesentery, including intestinal branches, in the same line as the visceral peritoneum incisions, starting at the level of the bowel wall. |
|  |  |  |  |
| 3. Small bowel resection | A. Bowel | Position | Position non-crushing clamps on the bowel, proximal and distal from the determined sites, to prevent leakage of bowel contents in the abdominal cavity. |
|  |  |  | Hazard - Clamp placement The clamp is solely placed on the bowel and not on the mesentery as this may compromise the vascularization of the bowel. |
|  |  | Transect | Transect the bowel at the determined sites. |
|  |  |  |  |
| 4. Small bowel anastomosis | A. Bowel corner mesenteric side | Close | Suture the first mesenteric corner with a double-armed suture through all layers from inside to outside and the second stitch with the same needle from outside to inside in the mesenteric corner of the opposite lumen using a monofilament slow absorbable thread. (Knot the suture but) do not cut it. |
|  | B. Bowel | Close | Continue suturing the posterior side of the anastomosis with this needle with an inside-outside-outside-inside technique. Use a continuous one layer technique with small parts of the mucosa and slightly larger parts of the seromuscular layer. Distance between the stitches should be around 3- 5 millimetre. The last stich should end on the outside. |
|  |  |  | TIP - Bowel turning If the other corner is reached at the mesenteric side, it is easiest to turn the bowel by turning the two clamps in such way that allows the surgeon to suture again towards him/her. |
|  | C. Bowel corner anti-mesenteric side | Close | After reaching the anti-mesenteric site of the posterior wall, switch to the other needle. The first stich should be made with the backhand from inside to outside. After this stich, the anterior site of the anastomosis can be closed from the mesenteric to the anti-mesenteric site. Finalise the sutures at the anti-mesenteric site with an adequate knot. |
|  |  |  | TIP - Two-layer anastomosing If a two-layer anastomosis is preferred the second layer is usually knotted with parts from the seromuscular layer only and using monofilament slowly absorbable thread (Lembert sutures). |
|  | D. Mesentery | Close | Close the mesentery after removal of the non-crushing clamps with standing absorbable sutures to prevent herniation of the bowel |
|  |  |  |  |
| 5. Abdominal wall closure | A. Linea alba | Approximate | Approximate the linea alba with slowly absorbable sutures with maximal steps of 1 cm at the time. Approximate the edges, but do not squeeze to prevent necrosis of the fascia. |
|  |  |  | HAZARD - Fascial necrosis While during closure of the fascia the sutures are tied too firmly, this will lead to necrosis. Note, a wound heals between the sutures. Sutures are placed to approximate the tissue and promote healing. |
|  | B. Skin | Close | Close the skin with running subcuticular absorbable monofilament sutures. |
|  |  |  | TIP - Peritonitis In case of peritonitis or higher risk of wound infection, the skin may be closed with standing non-absorbable monofilament sutures or skin staples. |
